# Structures and Stabilities of Carbon Chain Clusters Influenced by Atomic Antimony

**DOI:** 10.3390/molecules28031358

**Published:** 2023-01-31

**Authors:** Zhenjun Song, Xiji Shao, Wei Wu, Zhenzhong Liu, Meiding Yang, Mingyue Liu, Hai Wang

**Affiliations:** 1 School of Pharmaceutical and Chemical Engineering, Taizhou University, Taizhou 318000, China; 2 Department of Chemistry, Tongji University, Shanghai 200092, China; 3 School of Intelligent Engineering, Shaoguan University, Shaoguan 512005, China; 4 Department of Physics and Astronomy, London Centre for Nanotechnology, University College London, London WC1E 6BT, UK; 5 Research Institute of Zhejiang University-Taizhou, Zhejiang University, Taizhou 318000, China; 6 Key Laboratory of Yunnan Provincial Higher Education Institutions for Organic Optoelectronic Materials and Devices, Kunming University, Kunming 650214, China; 7 Yunnan Key Laboratory of Metal-Organic Molecular Materials and Devices, Kunming University, Kunming 650091, China

**Keywords:** carbon cluster, stability, antimony, parity effect, even-odd alteration

## Abstract

The C-C bond lengths of the linear magnetic neutral C*_n_*Sb, C*_n_*Sb^+^ cations and C*_n_*Sb^−^ anions are within 1.255–1.336 Å, which is typical for cumulene structures with moderately strong double-bonds. In this report, we found that the adiabatic ionization energy (*IE*) of C*_n_*Sb decreased with *n*. When comparing the *IE~n* relationship of C*_n_*Sb with that of pure C*_n_*, we found that the latter exhibited a stair-step pattern (*n* ≥ 6), but the *IE~n* relationship of C*_n_*Sb chains took the shape of a flat curve. The *IE*s of C*_n_*Sb were lower than those of corresponding pure carbon chains. Different from pure carbon chains, the adiabatic electron affinity of C*_n_*Sb does not exhibit a parity effect. There is an even-odd alternation for the incremental binding energies of the open chain C*_n_*Sb (for *n* = 1–16) and C*_n_*Sb^+^ (*n* = 1–10, when *n* > 10, the incremental binding energies of odd (*n*) chain of C*_n_*Sb^+^ are larger than adjacent clusters). The difference in the incremental binding energies between the even and odd chains of both C*_n_*Sb and pure C*_n_* diminishes with the increase in *n*. The incremental binding energies for C*_n_*Sb^−^ anions do not exhibit a parity effect. For carbon chain clusters, the most favorable binding site of atomic antimony is the terminal carbon of the carbon cluster because the terminal carbon with a large spin density bonds in an unsaturated way. The C-Sb bond is a double bond with Wiberg bond index (WBI) between 1.41 and 2.13, which is obviously stronger for a carbon chain cluster with odd-number carbon atoms. The WBI of all C-C bonds was determined to be between 1.63 and 2.01, indicating the cumulene character of the carbon chain. Generally, the alteration of WBI and, in particular, the carbon chain cluster is consistent with the bond length alteration. However, the shorter C-C distance did not indicate a larger WBI. Rather than relying on the empirical comparison of bond distance, the WBI is a meaningful quantitative indicator for predicting the bonding strength in the carbon chain.

## 1. Introduction

Small magnetic carbon clusters and related carbon-based materials have attracted much attention on the account of their important role in astrophysics, terrestrial processes, electronic spintronics, catalysis, and chemical engineering [1,2,3,4,5]. A deep understanding of the carbon nanostructure with adjustable bonding should facilitate the designing and synthesizing of size- and morphology-controlled carbon-based functional materials [6,7,8,9]. Previous theoretical and experimental studies have revealed that small carbon clusters are mainly of a linear structure [10,11,12]. The pure carbon clusters are ascertained to be intermediate in the production of diamonds, silicon carbine films, and a variety of chemical systems involving hydrocarbons [13,14,15]. Transition-metal doping for hybridization is an effective way to adjust the performance and electronic properties of nanomaterials [16,17,18,19,20]. In the interstellar medium, the reactivity of small carbon clusters is forfeited by quasi-collisionless conditions, and carbon atoms take the highly thermally stable, albeit highly reactive, form linear chains. When these metastable carbon chains encounter heteroatoms such as sulfur, oxygen, hydrogen, and nitrogen, they form more stable complexes.

Mass spectrometry is useful for detecting pure and doped carbon clusters with high stabilities. Theoretical studies of the linear carbon clusters show that doped by different heteroatom X, CnX/CnX^+^/CnX^−^ clusters have different parity effects in their stabilities [21,22,23]. Zhan and Iwata have used more sophisticated post-HF methods, including Møller-Plesset2 (MP2), MP4SDTQ, and the QCISD(T) method with different basis set to study the properties of CnN^-^ and CnP^−^ [24,25]. Zheng et al. produced cluster anions CnN^−^ and CnP^−^ from the laser ablation of appropriate samples and studied them by TOF mass spectrometry [26,27]. Small, doped carbon clusters have been extensively investigated theoretically and experimentally. Theoretical methods have been useful for studying heavier-atom-doped carbon clusters. Recently, theoretical work for lead [28], gallium [29], indium [30], gold [31], and tantalum [32] doped carbon clusters has been conducted. With the electronegativity modulation of heteroatom X, the parity effect in CnX may undergo dramatic changes. Their geometries, electronic structures, and bonding of small metal carbide clusters, such as antimony-doped carbon clusters, are yet to be determined systematically. In this paper, we report a density-functional-theory (DFT) study of the linear CnSb, CnSb^+^, and CnSb^−^ clusters.

## 2. Results and Discussion

### 2.1. Structural Optimization

**Figure 1:**
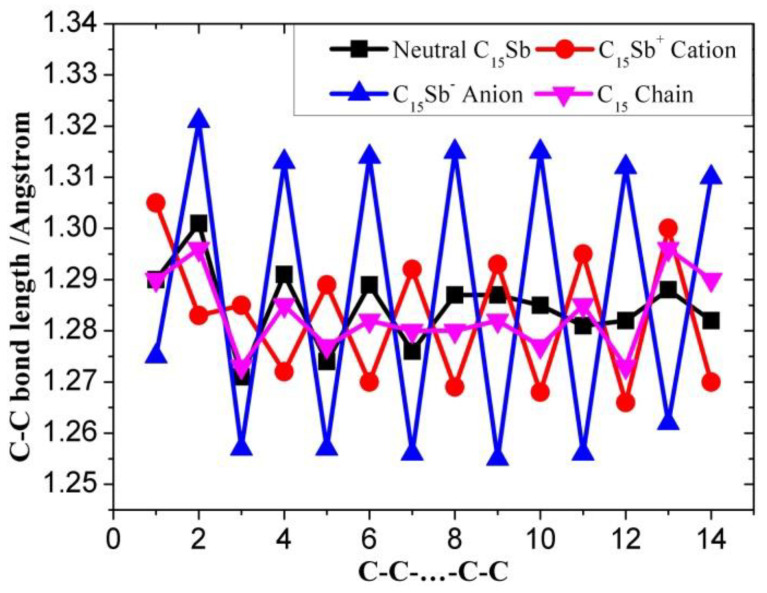
C-C bond length alteration of neutral C_15_Sb, C_15_Sb^+^ cation, C_15_Sb^−^ anion, and pure carbon chain C_15_ as a function of number of carbon atoms.

**Figure 2:**
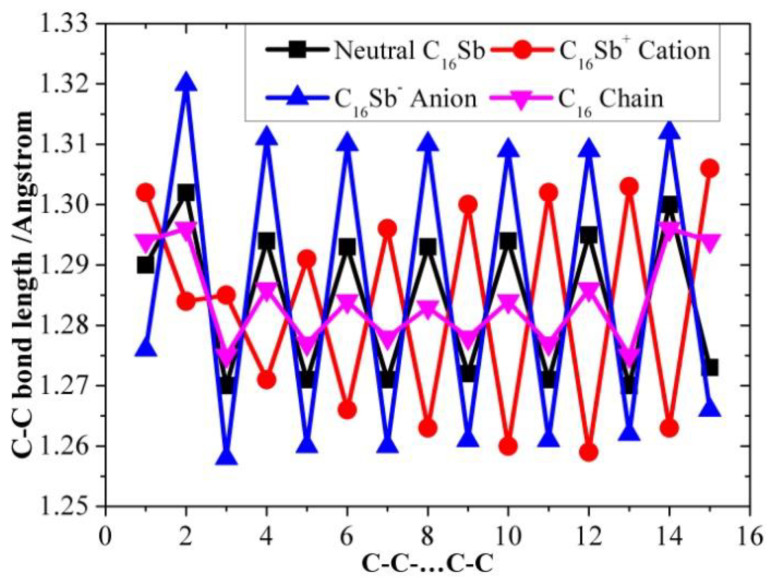
C-C bond length alteration of neutral C_16_Sb,C_16_Sb^+^ cation, C_16_Sb^−^ anion, and pure carbon chain C_16_ as a function of number of carbon atoms.

**Figure 3:**
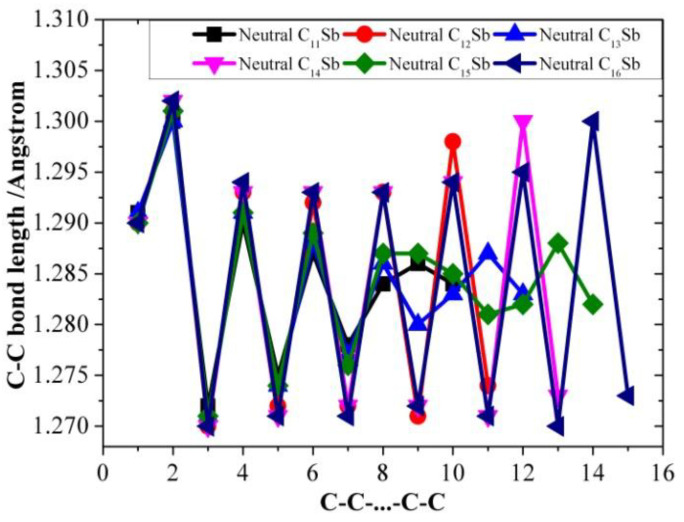
C-C bond length alteration of neutral C*_n_*Sb (*n* = 11–16) as a function of number of carbon atoms.

**Figure 4:**
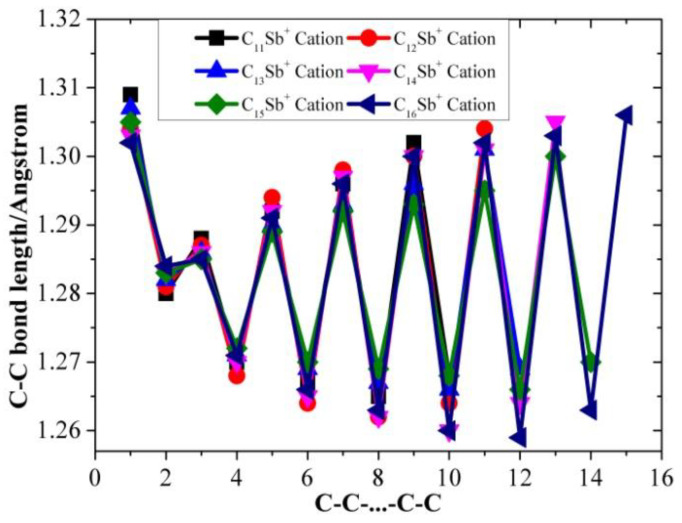
C-C bond length alteration of C*_n_*Sb^+^ cations (*n* = 11–16) as a function of number of carbon atoms.

**Figure 5:**
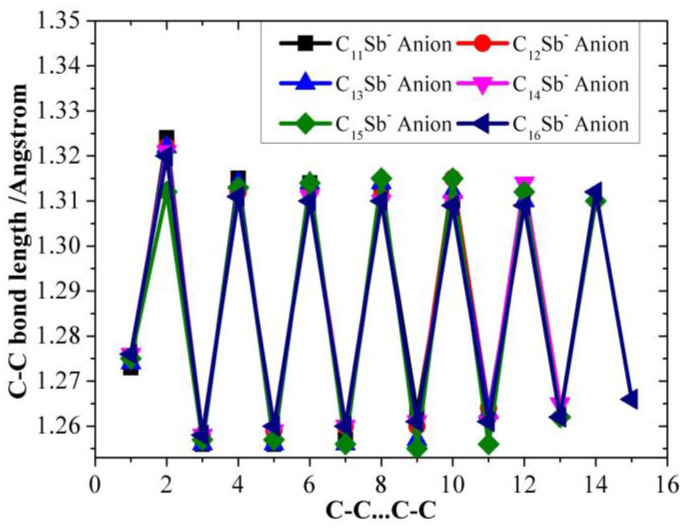
C-C bond length alteration of C*_n_*Sb^−^ anions (*n* = 11–16) as a function of number of carbon atoms.

**Figure 6:**
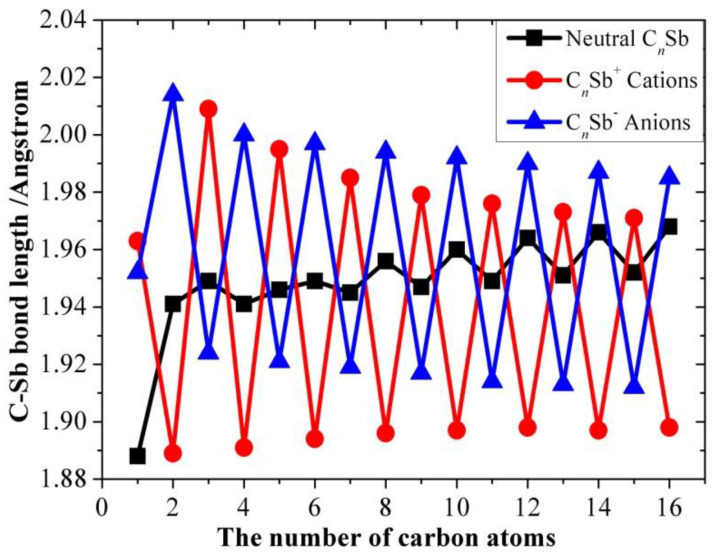
C-Sb bond length of neutral C*_n_*Sb, C*_n_*Sb^+^ cation, and C*_n_*Sb^−^ anion as a function of number of carbon atoms.

Table 1, Table 2 and Table 3 present the optimized bond lengths obtained at the B3LYP/6-31G(d) level, while Figure 1, Figure 2, Figure 3, Figure 4, Figure 5 and Figure 6 analyzed the C-C and C-Sb bond lengths. Table 4, Table 5 and Table 6 summarize the calculated total energies *E_n_* of optimized structures, differential energies Δ*E_n_*, binding energies BE, incremental binding energies Δ*E*^I^, dipole moments, and rotational constants. As shown in Figure 1, we can find that the C-C bond lengths of neutral C*_n_*Sb are within the range of 1.270–1.304 Å, exhibiting the character of both double-bonds (C-C bond length of ethylene is computed to be 1.331 nm by using B3LYP/6-31G(d)) and triple-bonds (C-C bond length of acetylene 1.205 Å by B3LYP/6-31G(d)); The C-Sb bond length of neutral C*_n_*Sb is within the range of 1.941 Å to 1.968 Å. The C-C bond length alteration (BLA) of neutral C*_n_*Sb is obvious and tends to be irregular in the vicinity of Sb. For C*_n_*Sb^+^ clusters, the C-C bond lengths are within a range from 1.259 Å to 1.334 Å, C-Sb bond lengths are in a range from 1.889 Å to 2.009 Å. The optimized BLA of corresponding C-C and C-Sb showed diverse tendencies compared with that of neutral C*_n_*Sb. The odd C*_n_*Sb^+^ chains also have a significant BLA effect; instead of being weakened and irregular C-C bond length, the C-C BLA near the antimony atom is slightly enhanced, which is markedly different from the corresponding neutral ones. The C-C bond lengths of C*_n_*Sb^−^ anions are within a range from 1.255 Å to 1.336 Å, and C-Sb bond lengths are within a range from 1.889 Å to 2.014 Å; the fluctuation trend of the BLA effect in even C*_n_*Sb^−^ anions have the same tendencies with that of neutral C*_n_*Sb; different from neutral C*_n_*Sb, odd C*_n_*Sb^−^ anions also have a distinct BLA effect, and this effect is more obvious than C*_n_*Sb^+^ cations. Unlike the C*_n_*Sb^+^ and C*_n_*Sb, the C-Sb bond lengths of even C*_n_*Sb^−^ anions are larger than the adjacent odd cluster anions, as depicted in Figure 5. We can preliminarily infer from C-C BLA and C-Sb BLA that the antimony atom of even C*_n_*Sb^+^ and odd C*_n_*Sb^−^ might combine with the carbon clusters more firmly than adjacent clusters (this speculation can be proved roughly right for small clusters by our calculation on their dissociation channels). When comparing the BLA of neutral and charged antimony-doped carbon chains with that of pure carbon chains, it is easy to deduce that through doping the antimony element, the properties of carbon clusters have been changed to a certain extent (Figure 1 and Figure 2). The C-C bond lengths are within the range of 1.255 Å–1.336 Å, typical for cumulene structures with moderately strong double-bonds. On the other hand, the clear alternation in C-C distances suggests that there is a substantial contribution of polyacetylenic valence-bond structures with an alternating triple and single C-C bond.

We mainly focused on the linear carbon chain clusters initially because many reported carbon clusters mainly adopt a linear shape as the ground state structure. For the antimony-doped larger carbon clusters (n > 10), we conducted density functional calculations to obtain the energies of ring structures. The total energies of C_11_Sb and C_14_Sb with a ring structure are 0.35 and 0.11 eV higher in energy than linear C_11_Sb and C_14_Sb clusters, while C_12_Sb, C_13_Sb, C_15_Sb, and C_16_Sb clusters with a ring structure are −0.14, −0.57, −0.53, and −0.83 eV lower in energy. Although the linear carbon cluster is not always the global minima in the potential surface, the linear carbon clusters possess a practical significance that the reactivity of carbon clusters increases dramatically with the increasing number of consecutive cumulene-like double bonds, and the linear carbon clusters can be formed in space-confined nanotube materials [33,34,35]. The linear carbon chain cluster with different lengths and doping atoms is a special allotrope type of carbon material different from graphite, diamond, graphene, carbon nanotubes, and fullerenes.

We made comparisons between 6-31G(d) and def2-TZVP basis sets for selected structural parameters, C-Sb stretching frequencies, and HOMO-LUMO gaps in Table 7. The C-C bonds adjacent antimony atoms of C_4_Sb, C_5_Sb, C_6_Sb, C_10_Sb, and C_11_Sb show only slight differences less than 0.011 Å, and the C-Sb bond distances are almost the same through using def2-TZVP and 6-31G(d) basis sets. C-Sb stretching frequencies tend to show negligible differences for the larger C_11_Sb and C_12_Sb. Although the C-Sb stretching frequency differences for C_4_Sb and C_5_Sb, it is slightly larger (6.2 cm^−1^ and 3.9 cm^−1^) under different basis sets, and we can clearly recognize that the theoretically predicted vibration modes are the same by comparing normal coordinates. The HOMO-LUMO gaps for C_4_Sb, C_5_Sb, C_6_Sb, C_10_Sb, and C_11_Sb are 1.27, 1.25, 1.17, 1.03, and 1.01 eV with 6-31G(d), while the HOMO-LUMO gaps are nearly identical with def2-TZVP basis set (1.26, 1.27, 1.16, 1.04, and 0.99 eV, respectively).

### 2.2. Electronic Configuration

The total symmetry of the pure carbon chain is $D_{\infty h}$ The core electron configuration for carbon is 1*s*^2^, and for antimony, 1*s*^2^2*s*^2^2*p*^6^3*s*^2^3*p*^6^3*d*^10^4*s*^2^4*p*^6^4*d*^10^. C*_n_*Sb has, therefore, 4*n* + 5 valence electrons. Based on molecular orbital theory, quantum chemical computations predict the electronic configurations of the linear ground-state C*_n_*Sb clusters as:

$C_{n}\mathrm{Sb}\{\begin{array}{c} \left(\mathrm{core}\right)1\sigma^{2}2\sigma^{2}1\pi^{4}3\sigma^{1},n=1\\ \left(\mathrm{core}\right)1\sigma^{2}2\sigma^{2}3\sigma^{2}\cdots (n+2)\sigma^{2}\\ \left(\mathrm{core}\right)1\sigma^{2}2\sigma^{2}3\sigma^{2}\cdots \left(n+2\right)\sigma^{2}n\pi^{2}\pi^{1},\;n\;\mathrm{is}\;\mathrm{even} \end{array}\frac{2n+1}{2}\pi^{2},\;n\;\mathrm{is}\;\mathrm{odd}$

Table 8 lists the valence orbital configuration of C*_n_*Sb (*n* = 1–16) clusters. C*_n_*Sb possesses (2*n* + 1) valence π-electrons and (2*n* + 4) valence σ-electrons. The (2*n* + 4) valence σ-electrons fully occupy the (*n* + 2) σ-orbitals. C*_n_*Sb has a ^…^π^1^ electronic configuration, ^2^Π state for *n*-even members and a ^…^π^3^ electronic configuration, ^2^Π state for *n*-odd members. C*_n_*Sb^+^ anions contain 2*n* valence π-electrons; even for *n*, these 2*n* π-electrons should fully populate *n* and doubly degenerate π-orbitals, resulting in a …π^4^ electronic configuration and a ^1^Σ^+^ state while for odd *n*, the highest occupied molecular orbital (HOMO) with π-symmetry is half-filled with two electrons, resulting in a ^…^π^2^ electronic configuration and a ^3^Σ^−^ state. For C*_n_*Sb^−^ anions, the situation is just the opposite. Two more π-electrons result in fully filled π-orbitals in *n*-odd C*_n_*Sb^−^ clusters and a half-filled π-orbital in *n*-odd C*_n_*Sb^−^ clusters.

**Figure 7:**
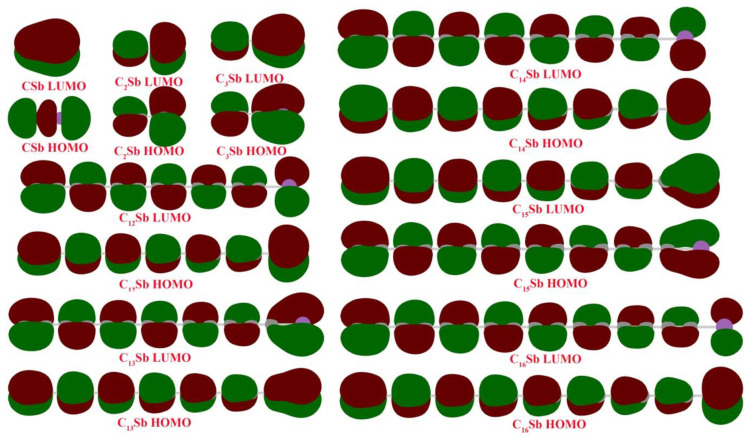
Highest occupied molecular orbitals (HOMO) and lowest unoccupied molecular orbitals (LUMO) for carbon chain clusters. The surface isovalue for molecular orbitals is 0.02.

The frontier molecular orbitals are depicted in Figure 7 with an isovalue of 0.02. Except for the CSb molecule, the LUMO of the other carbon chain clusters exhibits the same orbital shape as HOMO. The smallest CSb shows the σ-character of HOMO, while all the other C*_n_*Sb chain clusters show π-character HOMO with overlapping p orbitals shoulder to shoulder. For HOMO, the p orbital of the terminal carbon of the even-numbered carbon cluster always presents a different sign from the p orbital of antimony. While the p orbital of the terminal carbon of odd-numbered carbon cluster exhibits the same sign with a p orbital of antimony, it can form π bonding orbital with antimony. Therefore, the C-Sb bond of C*_n_*Sb with even-numbered carbon should be weaker than the C-Sb bond of C*_n_*_± 1_Sb with odd-numbered carbon. This result agrees well with the Wiberg bond index analysis that the C-Sb WBI of C*_n_*Sb with an even *n* tends to be smaller than the C-Sb WBI of C*_n_*Sb with odd *n*.

### 2.3. Electronic Properties

**Figure 8:**
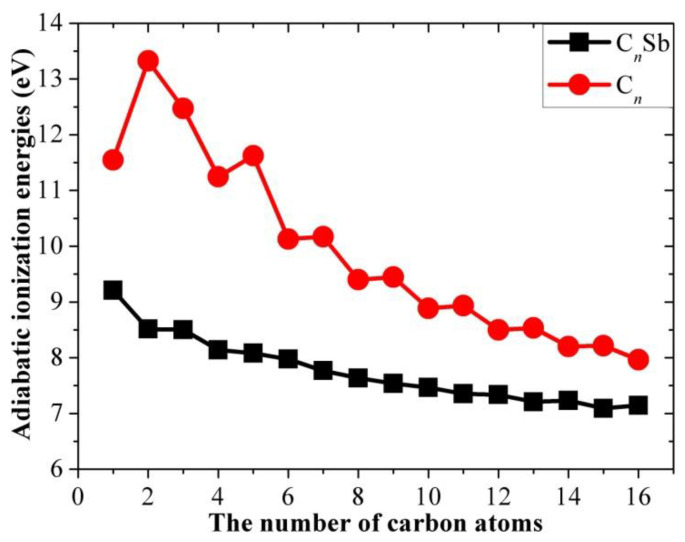
Adiabatic ionization energies of neutral C*_n_*Sb and pure carbon clusters C*_n_* as a function of the number of carbons.

As is known, the ion signal intensity in a mass spectrum is related to the electron affinity (EA) or ionization energies (IE, also called electron detachment energy) of the molecule. Thus, adiabatic ionization energies, defined as the energy required to remove an electron from the neutral clusters with a geometric change, are important parameters to understand the relative stability of the antimony-doped clusters with different sizes. Usually, there are three types of IE: Koopmans IE, vertical IE, and adiabatic IE. Koopmans IE is the HOMO energy, vertical IE is the energy difference between the neutral and ionic clusters at the neutral equilibrium geometry, and adiabatic IE is the energy difference between the neutral and ionic clusters at their respective equilibrium geometry (i.e., IE = E(optimized cation) − E(optimized neutral)). In this work, the adiabatic IE of C*_n_*Sb and C*_n_* clusters for their optimized structures were calculated and shown in Table 4 and Figure 8. As a whole, the ionization energies decrease with the size of the clusters, suggesting that larger C_n_Sb chains become less stable, e.g., when exposed to a strong electrical field or high temperature. As shown in Figure 8, the adiabatic IE of pure carbon chains C*_n_* has a stair-step shape (*n* ≥ 6), whereas C*_n_*Sb chains take the shape of a flat curve with a smaller gradient than pure carbon chains. The adiabatic IEs of the even and odd pure carbon chains (*n* ≥ 6) follow some nonlinear relationship; the IEs of carbon chains are larger than that of corresponding C*_n_*Sb, but the energy difference between them (IE_C*n*_-IE_C*n*Sb_) decreases when *n* increases. The electron affinity (EA) of C*_n_*Sb is defined as the energy released when an electron is attached to neutral C*_n_*Sb:

EA = *E*(optimized neutral) – *E*(optimized anion).

**Figure 9:**
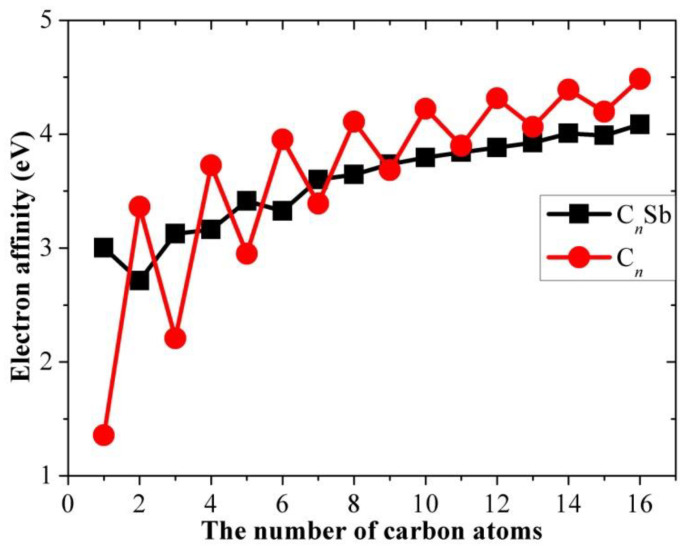
Adiabatic electron affinities of neutral C*_n_*Sb and pure carbon clusters C*_n_* as a function of the number of carbons.

This property is also related to the stability of the molecule in the TOF experiment and molecule. A higher EA means that more energy is released when an electron is added to a neutral molecule, and the generation of the corresponding anion is more readily performed. The calculated adiabatic EA data are presented in Table 4. In Figure 9, we compared the adiabatic EAs of C*_n_*Sb with that of pure carbon chains: the EAs of carbon chains have a clear alternation parity effect, the EAs of carbon chains with an even *n* are larger than the EAs of adjacent *n*-odd members but carbon chains doped with the antimony atom do not exhibit a parity effect. The computed EAs of the even and odd carbon chains followed a non-linear relationship with the number of carbons, respectively. The energy difference between EA_C*n*_ and EA_C*n*Sb_ was much smaller than (IE_C*n*_ - IE_C*n*Sb_), except when *n* = 1, and EA_C*n*_ was slightly larger than EA_C*n*Sb_ when *n* ≥ 10.

**Figure 10:**
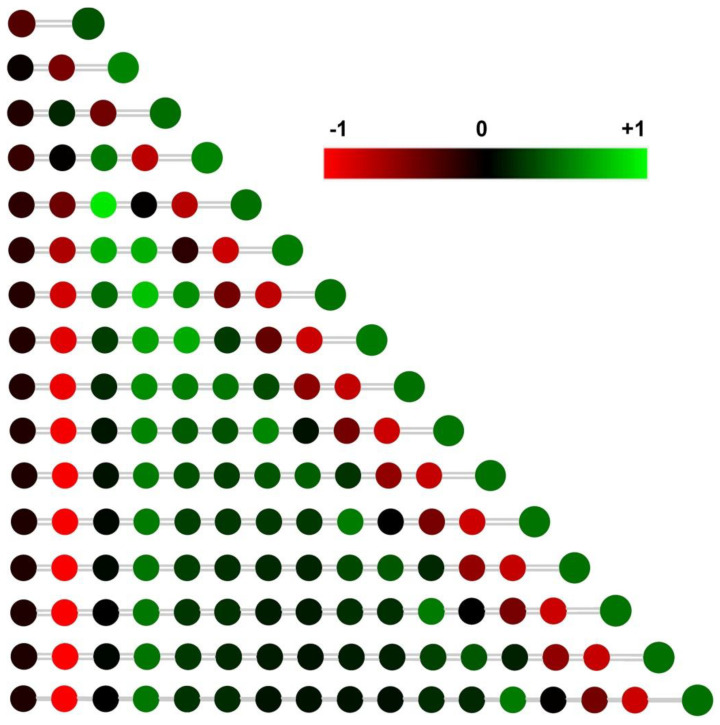
The NPA (natural population analysis) charge distribution for C*_n_*Sb chain clusters. The color range is depicted from −1 to 1. The small balls and the large balls in the right end indicate carbon and antimony atoms, respectively.

All the antimony atoms of C*_n_*Sb show positive charges, indicating that antimony denotes electrons to the carbon cluster. The antimony of CSb carries the least positive charge of 0.33 |e|, while the antimony of C_2_Sb carries the most positive charge of 0.52 |e|. Antimony atoms of larger C*_n_*Sb (*n* > 3) chain clusters show positive charges 0.43, 0.44, 0.49, 0.44, 0.48, 0.44, 0.46, 0.44, 0.46, 0.44, 0.45, 0.44, and 0.44 |e|, respectively. Thus, the carbon chain with an even-number of carbon atoms tends to exhibit higher antimony charges. The carbon atom nearest to antimony showed a negative charge due to the electron donation of antimony (Table 9). Without doping antimony, the two anomeric carbons at the right and the left end of the pure carbon chain show an identical charge value. With the antimony doping, the anomeric carbon shows significant charge differences (Table 9 and Figure 10). The charge differences between anomeric carbon atoms are calculated to be 0.43, 0.31, 0.51, 0.53, 0.62, 0.6, 0.65, 0.63, 0.66, 0.64, 0.67, 0.65, 0.67, 0.65, and 0.67 |e| for C*_n_*Sb (*n* = 2–16). This result suggests that the doping of antimony definitely changed the charge population of the carbon cluster.

**Figure 11:**
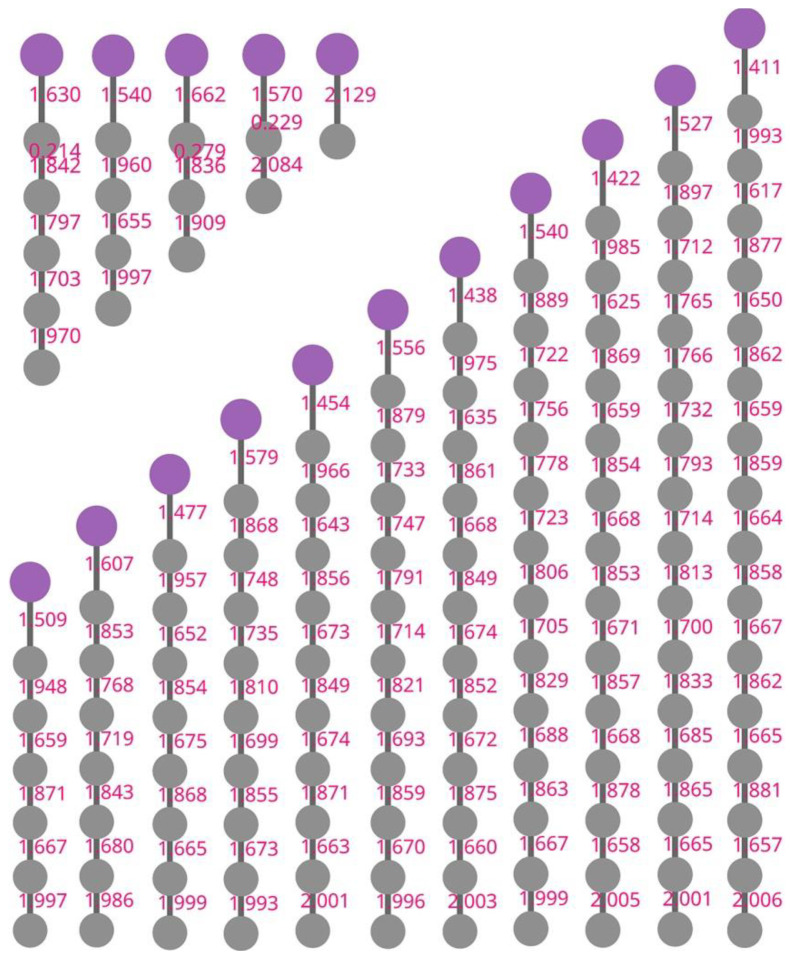
Computed Wiberg bond indices (WBI) for C*_n_*Sb.

The Wiberg bond indices of C*_n_*Sb are listed in Figure 11. The C-Sb bond is a double bond with WBI between 1.41 and 2.13, which is obviously stronger for a carbon chain cluster with odd-number carbon atoms (compared with neighboring carbon chain clusters with even-number carbon atoms). The WBI of all C-C bonds was determined to be between 1.63 and 2.01, indicating the cumulene character of the carbon chain. Generally, the alteration of WBI and, in particular, the carbon chain cluster is consistent with the bond length alteration. However, the shorter C-C distance did not indicate a larger WBI. For example, the largest WBI for C*_n_*Sb (*n* > 1) was calculated to be the terminal C-C bond index, while this terminal C-C bond was not the shortest C-C bond. Therefore, rather than relying on the empirical comparison of bond distance, the WBI is a meaningful quantitative indicator for predicting the bonding strength in the carbon chain.

### 2.4. Incremental Energies, Fragmental Energies, and Binding Sites

The relative stability of clusters can be also analyzed in terms of the energy differences between the neighboring size of the clusters, which is correlated with the “magic number” in cluster science [36]. Energy differences between C*_n_*Sb and C*_n−_*_1_Sb, C*_n_*Sb^+^ and C*_n_*_−1_Sb^+^, C*_n_*Sb^−^ and C*_n_*_−1_Sb^−^ (differential energies Δ*E_n_*, defined as Δ*E_n_ = E_n_* − *E_n−_*_1_) are listed in Table 5, Table 6 and Table 7, respectively. For the clusters with different sizes, the concept of the incremental binding energy, labeled as Δ*E*^I^, was introduced to compare their relative stabilities. As suggested by Pascoli and Lavendy [37,38], Δ*E*^I^ is just the reaction energy of the following processes:

C*_n_*Sb → C*_n−_*_1_Sb + C (DN1)

C*_n_*Sb^+^ → C*_n_*_−1_Sb^+^ + C (DC1)

C*_n_*Sb^−^ → C*_n_*_−1_Sb^−^ + C (DA1)

They can be computed as the consecutive binding energy (BE, atomization energy, listed in Table 5, Table 6 and Table 7) differences between the adjacent clusters,

Δ*E*^I^(C*_n_*Sb/C*_n_*Sb^+^/ C*_n_*Sb^−^) = BE(C*_n_*Sb/C*_n_*Sb^+^/C*_n_*Sb^−^) − BE(C*_n−_*_1_Sb/C*_n−_*_1_Sb^+^/C*_n−_*_1_Sb^−^)

where BE can be defined as the energy difference between a molecule and its component atoms:

BE(C*_n_*Sb/ C*_n_*Sb^+^/ C*_n_*Sb^−^) = *nE*(C) + *E*(Sb) − *E*(C*_n_*Sb/ C*_n_*Sb^+^/ C*_n_*Sb^−^)

**Figure 12:**
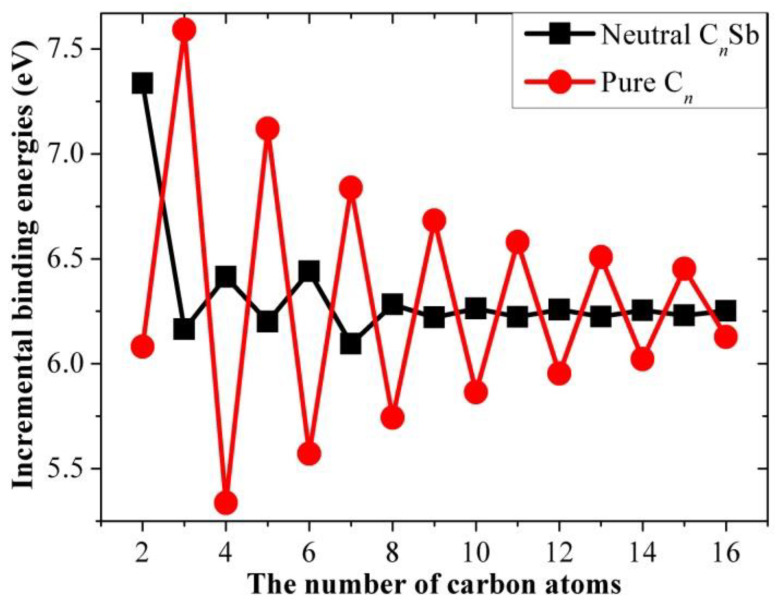
Increment binding energies of C*_n_*Sb and pure C*_n_* chains as a function of the number of carbon atoms.

**Figure 13:**
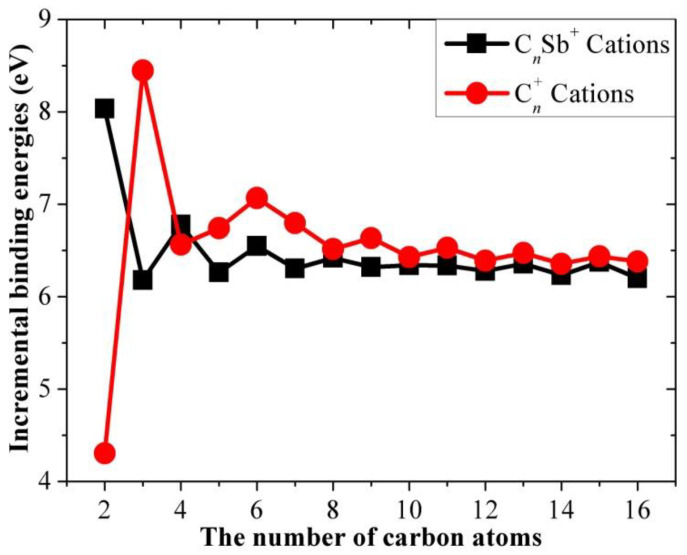
Incremental binding energies of C*_n_*Sb^+^ cations and pure C*_n_* cations as a function of the number of carbon atoms.

**Figure 14:**
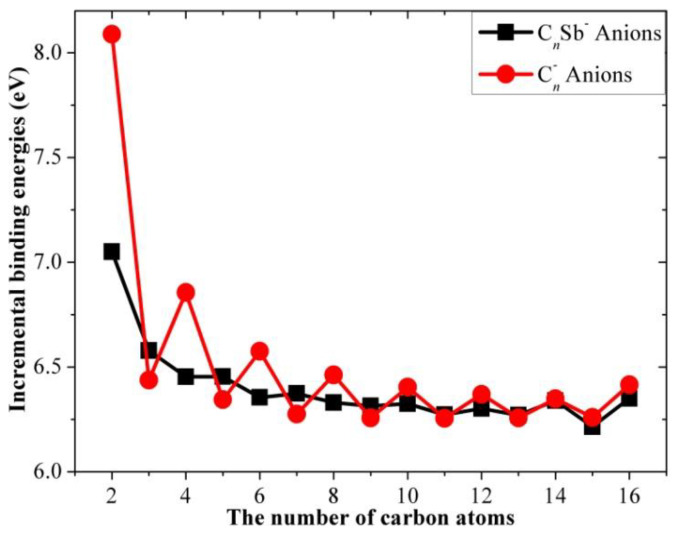
Incremental binding energies of C*_n_*Sb^−^ anions and pure C*_n_*^−^ anions.

The results for the incremental binding energy as a function of the number of carbon atoms for the different open-chain C*_n_*Sb/C*_n_*Sb^+^/C*_n_*Sb^−^ clusters and pure carbon chains are shown in Figure 12, Figure 13 and Figure 14. It can be observed that there is an even-odd alternation for the open chain C*_n_*Sb and C*_n_*Sb^+^ (*n* = 1–10, when *n* > 10, the parity effect is less obvious and *n*-odd members of C*_n_*Sb^+^ are slightly larger), with even species being comparatively more stable than odd ones; the parity variation tendency of neutral C*_n_*Sb is opposite to that of pure C*_n_* (i.e., *n*-even carbon chains are less stable than adjacent *n*-odd ones) and the variation amplitude is much smaller than pure C*_n_*. The difference in Δ*E*^I^ between the even and odd species of both C*_n_*Sb and pure C*_n_* chains diminishes with the increase in carbon atoms; different from C*_n_*^-^ anions, the Δ*E*^I^ for C*_n_*Sb^−^ anions did not exhibit a parity effect. However, these patterns of Δ*E*^I^ for C*_n_*Sb^+^ and C*_n_*Sb^−^ cannot be simply explained by the “valence π-electron number” rule.

**Figure 15:**
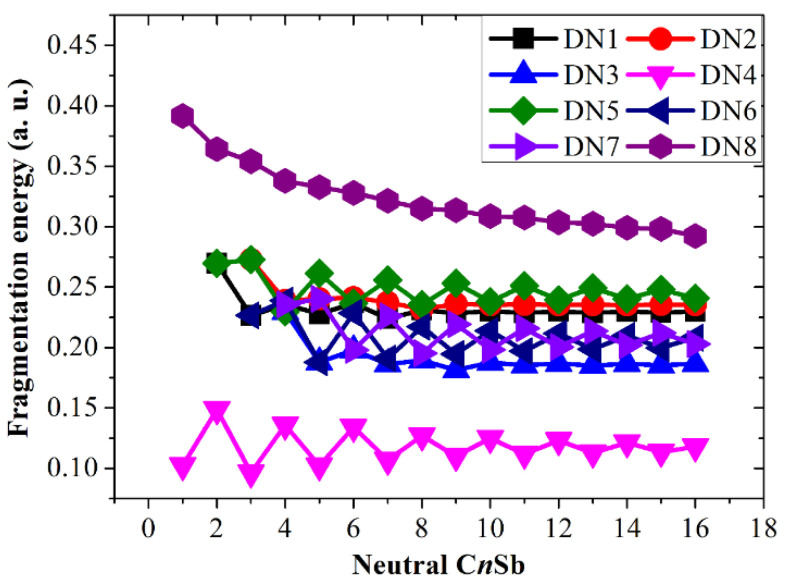
Fragmentation energies of neutral C*_n_*Sb clusters. DNx corresponds to different dissociation channels, as shown in the text.

**Figure 16:**
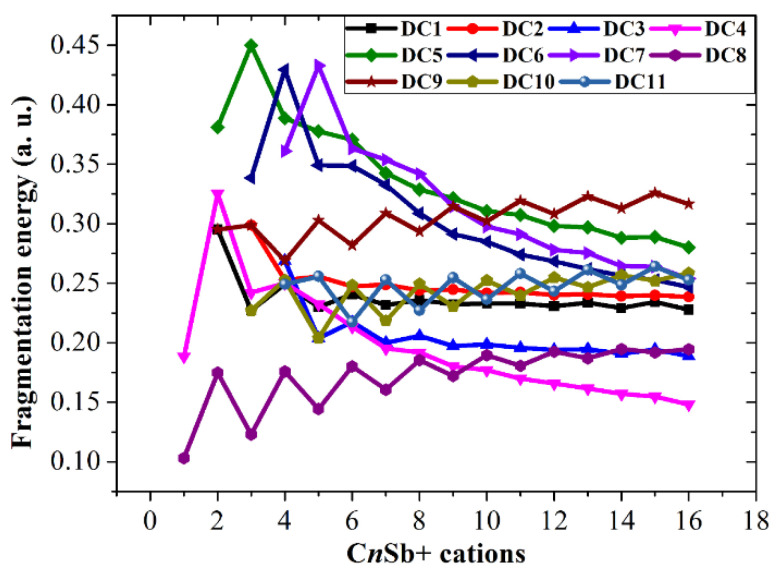
Fragmentation energies of C*_n_*Sb^+^ cations. DC*x* corresponds to different dissociation channels, as shown in the text.

**Figure 17:**
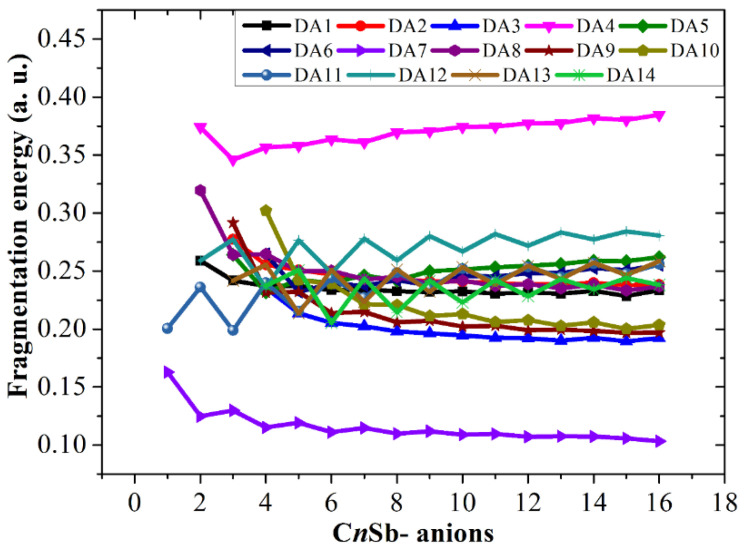
Fragmentation energies of C_n_Sb^−^ anions. DA*x* corresponds to different dissociation channels, as shown in the text.

The fragmentation energies accompanying channels DN1, DC1, and DA1, i.e., △*E*^I^, have been discussed. In addition, the fragmentation energies for many other dissociation reactions are calculated and exhibited in Figure 15, Figure 16 and Figure 17, including the following seven channels for neutral C*_n_*Sb clusters:

C*_n_*Sb → C*_n_*_−2_Sb + C_2_(DN2)

C*_n_*Sb → C*_n_*_−3_Sb + C_3_ (DN3)

C*_n_*Sb → C*_n_* + Sb (DN4)

C*_n_*Sb → C*_n_*_−1_ + CSb (DN5)

C*_n_*Sb → C*_n_*_−2_ + C_2_Sb (DN6)

C*_n_*Sb → C*_n_*_−3_ + C_3_Sb (DN7)

C*_n_*Sb → C*_n_*^-^ + Sb^+^ (DN8)

The following ten channels for cationic CnSb+ cations:

C*_n_*Sb^+^ → C*_n_*_−2_Sb^+^ + C_2_ (DC2)

C*_n_*Sb^+^ → C*_n_*_−3_Sb^+^ + C_3_ (DC3)

C*_n_*Sb^+^ → C*_n_*^+^ + Sb (DC4)

C*_n_*Sb^+^ → C*_n_*_−1_^+^ + CSb (DC5)

C*_n_*Sb^+^ → C*_n_*_−2_^+^ + C_2_Sb (DC6)

C*_n_*Sb^+^ → C*_n_*_−3_^+^ + C_3_Sb (DC7)

C*_n_*Sb^+^ → C*_n_* + Sb^+^ (DC8)

C*_n_*Sb^+^ → C*_n_*_−1_ + CSb^+^ (DC9)

C*_n_*Sb^+^ → C*_n_*_−2_ + C_2_Sb^+^ (DC10)

C*_n_*Sb^+^ → C*_n_*_−3_ + C_3_Sb^+^ (DC11)

and the following thirteen channels for anionic C*_n_*Sb^−^ anions:

C*_n_*Sb^−^ → C*_n_*_−2_Sb^−^ + C_2_ (DA2)

C*_n_*Sb^−^ → C*_n_*_−3_Sb^−^ + C_3_ (DA3)

C*_n_*Sb^−^ → C*_n_*_−1_Sb + C^−^ (DA4)

C*_n_*Sb^−^ → C*_n_*_−2_Sb + C_2_^−^ (DA5)

C*_n_*Sb^−^ → C*_n_*_−3_Sb + C_3_^−^ (DA6)

C*_n_*Sb^−^ → C*_n_*^−^ + Sb (DA7)

C*_n_*Sb^−^ → C*_n_*_−1_^−^+ CSb (DA8)

C*_n_*Sb^−^ → C*_n_*_−2_^−^+ C_2_Sb (DA9)

C*_n_*Sb^−^ → C*_n_*_−3_^−^+ C_3_Sb (DA10)

C*_n_*Sb^−^ → C*_n_* + Sb^−^ (DA11)

C*_n_*Sb^−^ → C*_n_*_−1_ + CSb^−^ (DA12)

C*_n_*Sb^−^ → C*_n_*_−2_ + C_2_Sb^−^ (DA13)

C*_n_*Sb^−^ → C*_n_*_−3_ + C_3_Sb^−^ (DA14)

These channels can be divided into four types: (1) losing neutral small carbon particles such as C, C_2_, or C_3_; (2) losing neutral antimony-contained small fragments such as Sb, CSb, C_2_Sb, or C_3_Sb; (3) losing ionic defects, such as Sb^+^/Sb^−^, CSb^+^/CSb^−^, C_2_Sb^+^/C_2_Sb^−^, or C_3_Sb^+^/C_3_Sb^−^ fragments (for C*_n_*Sb^+^/C*_n_*Sb^−^); and (4) losing anionic carbons, such as C^−^, C_2_^−^, and C_3_^−^ fragments (only for C*_n_*Sb^−^). Comparing the fragmentation energies can help us to find some dominant channels for each kind of cluster in the discussion.

**Figure 18:**
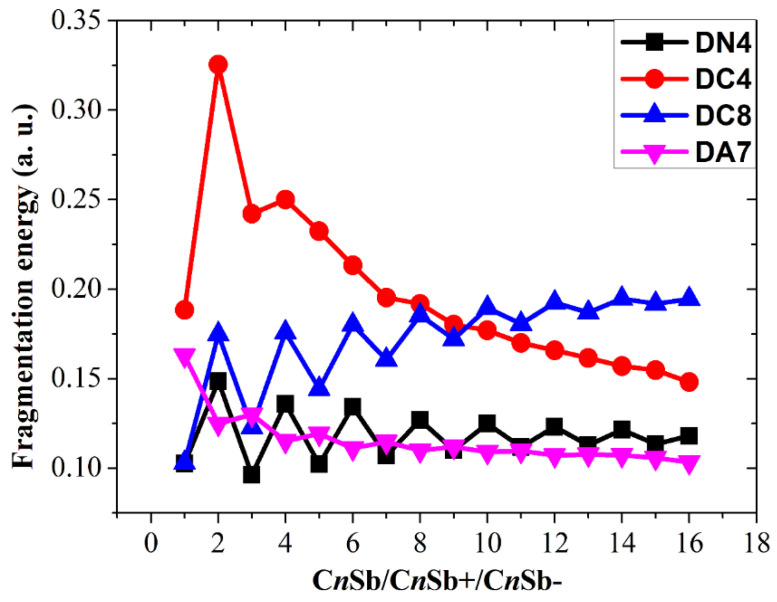
The most favorable dissociation channels for C_n_Sb/ C_n_Sb^+^ / C_n_Sb^−^.

The fragmentation energies of channels DN1, DC1, and DA1 are also included for comparison. It is clear that losing an antimony atom is the dominant channel for neutral C*_n_*Sb (channel DN4). For small C*_n_*Sb^+^ cations (*n* = 1–9), the most favorable fragmentation channel is the loss of the Sb^+^ ion (channel DC8). However, when *n* ≥ 10, losing an antimony atom (DC4) becomes the dominant channel. The most favorable dissociation pathway for CSb^−^ and C_n_Sb^−^ (*n* = 2–16) is the loss of the Sb^−^ ion and the loss of the antimony atom, respectively. The most favorable dissociation channels for C*_n_*Sb/C*_n_*Sb^+^/C*_n_*Sb^−^ are illustrated in Figure 18, from which we can draw some conclusions: the fragmentation energies for DN4 exhibit a parity effect, i.e., the even C*_n_*Sb clusters are more stable while the odd C*_n_*Sb clusters are easy to dissociate; the fragmentation energies for DC8 (*n* = 1–9) also showed an alternation effect with the same alternation trend as DN4. the fragmentation energies for DC4 did not show fluctuation or a decrease monotonically as the *n* number rose; when n > 4 C*_n_*Sb^+^, is more stable than C*_n_*Sb^−^ anions and neutral C*_n_*Sb. The subtle alternation for DA7 exists when *n* ≤ 10; when *n* > 10, the C*_n_*Sb^−^ anion is in its least stable form compared with C*_n_*Sb and C*_n_*Sb^+^.

**Figure 19:**
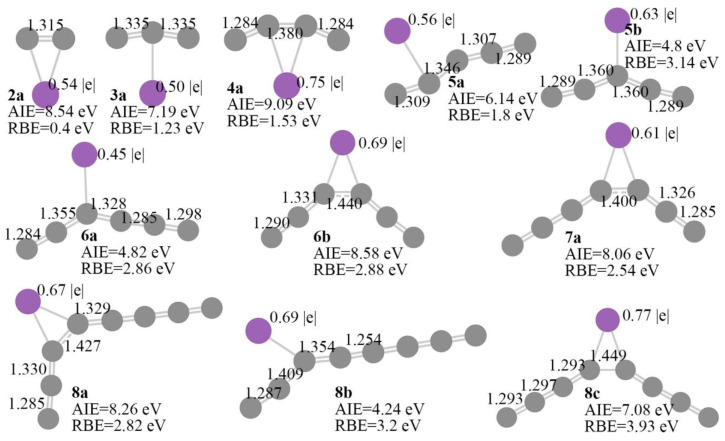
Structural geometry, adiabatic ionization energy (AIE), relative binding energy (RBE), and charge population influenced by binding site of antimony atom. Relative binding energy is calculated by equation: $\mathrm{RBE}=E_{b}\left(\mathrm{nt}\right)-E_{b}\left(t\right)$, where $E_{b}\left(\mathrm{nt}\right)$ and $E_{b}\left(t\right)$ stand for structure with antimony binding on nonterminal carbon and linear structure with antimony binding on terminal carbon, respectively. Notations na, nb, and nc represent the structures with *n* carbon atoms.

For carbon chain clusters, the most favorable binding site of atomic antimony is the terminal carbon of the carbon cluster because the terminal carbon with a large spin density bonds in an unsaturated way. We have constructed initial structures with antimony binding on nonterminal carbon and found that the optimization of these structures often encounters a convergence problem or loop back to the linear structure with antimony binding to the terminal carbon. For the structures which encounter convergence problems or converge to geometry with antimony binding on nonterminal carbon, we further optimized them and obtained the local minima (Figure 19). For antimony binding on C_2_, the 2a nonlinear structure showed a C-C distance of 1.315 Å, longer than the linear structure. The results indicate that the construction of the nonlinear shape C*_n_*Sb can further activate the C-C bond. Nonlinear C_3_Sb and C_4_Sb show C_2V_ symmetric structures. The C-C bonds of C_3_Sb are all 1.335 Å, while the C-C bond adjacent to antimony shows a substantially longer distance of 1.380 Å. Nonlinear C_5_Sb with antimony binding on the second or third carbon does not exhibit bond length alteration. Nonlinear C_6_Sb and C_7_Sb with antimony binding on the third carbon or binding on the bridge site between the third and fourth carbon do not exhibit bond length alteration effects. The C-C bond distances adjacent to antimony are calculated to be 1.346 and 1.360 Å for 5a and 5b, respectively, which are much longer than 1.288 Å for linear C_5_Sb. The C-C bond distances adjacent to the antimony of 6a and 6b are calculated to be 1.355 and 1.440 Å, which is much longer than 1.277 Å for the linear carbon chain C_6_Sb. The bond length alteration does not exist for nonlinear C_7_Sb. The antimony binding on the second or third carbon of C_7_ leads to the reconstruction and formation of the linear carbon chain. The bond length alteration effect exists in unstable nonlinear C_8_Sb (8c) with s high RBE of 3.93 eV. Structures 8a and 8b do not show the bond length alteration effect.

For the low energy minia of nonlinear C_2_Sb to C_8_Sb, the adiabatic ionization energies were calculated to be 8.54, 7.19, 9.09, 6.14, 4.82, 8.06, and 8.26 eV, respectively, indicating that the nonlinear C_5_Sb and C_6_Sb could be more easily ionized. Comparatively, the linear carbon chains C*_n_*Sb show more constant adiabatic ionization energy with the increase in the carbon number. The relative binding energies of nonlinear C_2_Sb to C_8_Sb with antimony binding on the sides of carbon clusters (relative to the binding energy of the linear chain cluster) are calculated to be 0.4, 1.23, 1.53, 1.8, 2.86, 2.54, and 2.82 eV, respectively. The results indicate that the relative binding energy is significantly large except for the much smaller carbon cluster C_2_Sb. Quantitatively, the binding energy of nonlinear C_2_Sb to C_8_Sb with antimony binding on the sides of carbon clusters were calculated to be −3.33, −3.24, −1.85, −2.64, −0.34, −1.52, and −0.28 eV, respectively. Antimony is a metallic element exhibiting low electron affinity. If the antimony atom is changed to nitrogen with strong electronegativity, we can test the structural geometry, adiabatic ionization energy, and natural charge population of C_6_N and C_7_N. The bond length alteration effect of C_6_N and C_7_N is much different from that in C_6_Sb and C_7_Sb. The bond length difference between the adjacent C-C bonds is larger than that in the antimony-doped carbon chain cluster. The C-C bond adjacent to nitrogen is substantially longer than that adjacent to antimony. The adiabatic ionization energies of C_6_N and C_7_N were calculated to be 8.82 and 9.17 eV, which are larger than the C_6_Sb and C_7_Sb, indicating the higher stability of the nitrogen-doped carbon chain. Different from the charging state of antimony, the natural charge population analysis of C_6_N and C_7_N indicates that nitrogen atoms are both negatively charged with −0.42 |e|.

## 3. Computational Methods

To explore the structure and energetics in the linear antimony-doped carbon clusters, full geometry optimizations were performed using density-functional theory methods implemented in the Gaussian 03 program [39]. The Molecular mechanic’s algorithm is frequently employed to investigate very large carbon-based materials because of the efficiency in predicting the binding and delivery mechanism. Density-functional calculations are verified to be effective and have an accurate strategy to reveal the geometric structures [40,41] and electronic properties of various nanomaterials [42,43,44,45]. The B3LYP exchange-correlation function consists of Fock’s exact exchange and Beck’s three-parameter nonlocal exchange function, along with the nonlocal correlation function developed by Lee et al. [46]. B3LYP was chosen here because the previous research suggests that hybrid-functional is reliable and highly efficient for molecules and clusters [47,48], while the post-HF Ab initio method is accurate but time-consuming [49,50]. A medium-size basis set 6-31G(d) was used for a carbon atom, and the Stuttgart/Bonn relativistic effective core potential (SDD) basis set was used for antimony. The geometries and relative energies of heteroatom-doped carbon clusters obtained with the B3LYP method were very close to those with the coupled cluster single and double (triple) (CCSD(T)) and QCISD(T) method [51,52]. Vibrational frequencies were computed at the same level using a harmonic approximation to assess the nature of the optimized structures. Zero-point energies (ZPE) were evaluated as well using the same methodology. The optimized structures were then used for single-point calculations at the B3LYP/6-311++G(3df,3pd) level. In the computation, bond lengths of the magnetic neutral CnSb, CnSb^+^ cations, and CnSb^−^ anions (*n* = 1–16) clusters have been optimized through the use of B3LYP methods with a 6-31G(d) basis set. Subsequently, the corresponding harmonic vibrational frequencies are evaluated at the same level. To further verify the reliability of the optimized geometries, we have carefully checked every computation step that might cause possible numerical calculation errors. We have adopted the default convergence criteria for self-consistent-field (SCF) calculation, i.e., 10^−8^ for the root mean square density and 10^−6^ for the maximum density, and for geometry optimization, 0.00030 This includes the Hartree/Bohr radius for the root mean square force and 0.00045 Hartree/Bohr radius for the maximum force. In addition, the convergence criteria for the energy change in the final step of geometry optimization was set to be 10^−7^ Hartree.

## 4. Conclusions

The linear carbon chain cluster with different lengths and doping atoms is a special allotrope type of carbon material different from graphite, diamond, graphene, carbon nanotubes, and fullerenes. We have conducted a systematic DFT study on linear C*_n_*Sb/C*_n_*Sb^+^/C*_n_*Sb^−^ clusters with sizes of *n* = 1–16 and compared these with pure C*_n_* clusters. C-C bond lengths of the linear neutral C*_n_*Sb, C*_n_*Sb^+^ cations and C*_n_*Sb^−^ anions are within 0.1255−0.1336 nm, which is typical of cumulene structures with moderately strong double bonds. However, the alternation in C-C distances suggests that there is a substantial contribution of polyacetylenic valence-bond structures with an alternating triple and single C-C bond. The C-C BLA of neutral CnSb is obvious for *n*-even clusters, and for *n*-odd clusters, the BLA tends to be irregular in the vicinity of Sb. We can deduct from C-C BLA and C-Sb BLA that the antimony atom of *n*-odd C_n_Sb^−^ anions and *n*-even C*_n_*Sb^+^ (*n* = 1–10) cations are combined more firmly than adjacent clusters. This is roughly proved to be right by our calculation of their dissociation channels. When comparing the BLA of neutral and charged antimony-doped carbon chains to that of pure carbon chains, it is easy to deduce that through doping the antimony element, the properties of carbon clusters are changed. The adiabatic IE of C*_n_*Sb decreased with the rise in the *n* number, suggesting that larger C*_n_*Sb chains become less stable. When comparing the IE of C*_n_*Sb with that of pure C*_n_*, we found that the latter had a stair-step pattern (*n* ≥ 6), but C*_n_*Sb chains took the shape of a flat curve. The IEs of carbon chains are larger than that of corresponding C*_n_*Sb, but the energy difference (IE_C*n*_-IE_C*n*Sb_) decreases with increasing *n*. Different from pure carbon chains, the adiabatic electron affinity of C*_n_*Sb do not exhibit a parity effect. There is an even-odd alternation for △*E*^I^ of the open chain C*_n_*Sb (*n* = 1–16) and C*_n_*Sb^+^ (*n* = 1–10, when *n* > 10, △*E*^I^ of *n*-odd members of C*_n_*Sb^+^ are larger), with the *n*-even species being comparatively more stable than *n*-odd ones. The △*E*^I^ for C*_n_*Sb^−^ anions does not exhibit a parity effect. For carbon chain clusters, the most favorable binding site of atomic antimony is the terminal carbon of the carbon cluster because the terminal carbon with a large spin density bonds in an unsaturated way. The C-Sb bond is a double bond with WBI between 1.41 and 2.13, which is obviously stronger for a carbon chain cluster with odd-number carbon atoms (compared with neighboring carbon chain clusters with even-number carbon atoms). The WBI of all C-C bonds was determined to be between 1.63 and 2.01, indicating the cumulene character of the carbon chain. Generally, the alteration of WBI and, in particular, the carbon chain cluster is consistent with the bond length alteration. However, the shorter C-C distance did not indicate a larger WBI. For example, the largest WBI for C*_n_*Sb (*n* > 1) was calculated to be the terminal C-C bond index, while this terminal C-C bond was not the shortest C-C bond. Therefore, rather than relying on the empirical comparison of bond distance, the WBI is a meaningful quantitative indicator for predicting the bonding strength in the carbon chain. For HOMO, the p orbital of the terminal carbon of the even-numbered carbon cluster always presents a different sign from the p orbital of antimony. While the p orbital of the terminal carbon of odd-numbered carbon cluster exhibits the same sign with a p orbital of antimony, it can form π bonding orbital with antimony. Therefore, the C-Sb bond of C*_n_*Sb with even-numbered carbon should be weaker than the C-Sb bond of C*_n_*_±1_Sb with odd-numbered carbon. This result agrees well with the Wiberg bond index analysis.

## Figures and Tables

**Table 1:** Optimized bond lengths (Å) for neutral C*_n_*Sb at b3lyp/6-31G(d) theoretical level.

CSb	1.888															
C2Sb	1.300	1.941														
C3Sb	1.301	1.296	1.949													
C4Sb	1.291	1.304	1.277	1.941												
C5Sb	1.294	1.297	1.280	1.288	1.946											
C6Sb	1.291	1.301	1.271	1.295	1.277	1.949										
C7Sb	1.292	1.299	1.274	1.288	1.282	1.286	1.945									
C8Sb	1.291	1.301	1.271	1.293	1.272	1.296	1.276	1.956								
C9Sb	1.291	1.300	1.272	1.289	1.276	1.285	1.284	1.285	1.947							
C10Sb	1.290	1.301	1.270	1.293	1.272	1.292	1.272	1.297	1.275	1.960						
C11Sb	1.291	1.300	1.272	1.290	1.275	1.287	1.278	1.284	1.286	1.284	1.949					
C12Sb	1.290	1.301	1.270	1.293	1.272	1.292	1.272	1.293	1.271	1.298	1.274	1.964				
C13Sb	1.291	1.300	1.271	1.291	1.274	1.288	1.277	1.286	1.280	1.283	1.287	1.283	1.951			
C14Sb	1.290	1.302	1.270	1.293	1.271	1.293	1.272	1.293	1.272	1.294	1.271	1.300	1.273	1.966		
C15Sb	1.290	1.301	1.271	1.291	1.274	1.289	1.276	1.287	1.278	1.285	1.281	1.282	1.288	1.282	1.952	
C16Sb	1.290	1.302	1.270	1.294	1.271	1.293	1.271	1.293	1.272	1.294	1.271	1.295	1.270	1.300	1.273	1.968

**Table 2:** Optimized bond length (Å) for C*_n_*Sb^+^ cations at b3lyp/6-31G(d) theoretical level.

CSb	1.963															
C2Sb	1.326	1.889														
C3Sb	1.334	1.270	2.009													
C4Sb	1.315	1.277	1.300	1.891												
C5Sb	1.321	1.272	1.302	1.264	1.995											
C6Sb	1.311	1.276	1.291	1.268	1.301	1.894										
C7Sb	1.315	1.276	1.293	1.264	1.303	1.265	1.985									
C8Sb	1.308	1.278	1.290	1.266	1.295	1.266	1.303	1.896								
C9Sb	1.311	1.278	1.290	1.268	1.295	1.264	1.302	1.267	1.979							
C10Sb	1.306	1.279	1.289	1.267	1.295	1.263	1.298	1.265	1.304	1.897						
C11Sb	1.309	1.280	1.288	1.270	1.292	1.267	1.296	1.265	1.302	1.268	1.976					
C12Sb	1.304	1.281	1.287	1.268	1.294	1.264	1.298	1.262	1.300	1.264	1.304	1.898				
C13Sb	1.307	1.282	1.286	1.271	1.290	1.269	1.293	1.267	1.296	1.266	1.301	1.269	1.973			
C14Sb	1.303	1.283	1.286	1.270	1.292	1.265	1.297	1.262	1.300	1.260	1.301	1.264	1.305	1.897		
C15Sb	1.305	1.283	1.285	1.272	1.289	1.270	1.292	1.269	1.293	1.268	1.295	1.266	1.300	1.270	1.971	
C16Sb	1.302	1.284	1.285	1.271	1.291	1.266	1.296	1.263	1.300	1.260	1.302	1.259	1.303	1.263	1.306	1.898

**Table 3:** Optimized bond length (Å) for C*_n_*Sb^−^ anions at b3lyp/6-31G(d) theoretical level.

CSb	1.952															
C2Sb	1.280	2.014														
C3Sb	1.278	1.326	1.924													
C4Sb	1.273	1.336	1.260	2.000												
C5Sb	1.273	1.326	1.262	1.314	1.921											
C6Sb	1.273	1.329	1.256	1.323	1.261	1.997										
C7Sb	1.272	1.326	1.256	1.315	1.263	1.311	1.919									
C8Sb	1.273	1.326	1.257	1.317	1.258	1.319	1.262	1.994								
C9Sb	1.273	1.325	1.256	1.316	1.257	1.313	1.264	1.310	1.917							
C10Sb	1.274	1.324	1.257	1.314	1.259	1.314	1.259	1.317	1.263	1.992						
C11Sb	1.273	1.324	1.256	1.315	1.256	1.314	1.257	1.312	1.263	1.310	1.914					
C12Sb	1.275	1.322	1.258	1.312	1.259	1.312	1.260	1.311	1.260	1.315	1.264	1.990				
C13Sb	1.274	1.322	1.256	1.314	1.256	1.314	1.256	1.314	1.257	1.312	1.263	1.310	1.913			
C14Sb	1.276	1.321	1.258	1.312	1.259	1.311	1.260	1.310	1.261	1.310	1.261	1.314	1.265	1.987		
C15Sb	1.275	1.321	1.257	1.313	1.257	1.314	1.256	1.315	1.255	1.315	1.256	1.312	1.262	1.310	1.912	
C16Sb	1.276	1.320	1.258	1.311	1.260	1.310	1.260	1.310	1.261	1.309	1.261	1.309	1.262	1.312	1.266	1.985

**Table 4:** The total energies *E_n_*, differential energies Δ*E_n_*, incremental energies Δ*E*^I^, binding energies BE, adiabatic ionization energies IE, adiabatic electron affinities EA, dipole moments, and rotational constants (RC) of neutral C*_n_*Sb.

C*_n_*Sb	*E_n_* (a. u.)	Δ*E_n_* (a. u.)	BE (a. u.)	Δ*E*^I^ (kcal/mol)	IE (kcal/mol)	EA (kcal/mol)	μ (debye)	RC (MHz)
1	−43.35455		0.10241		212.4	69.2	3.13	12990
2	−81.48162	−38.12707	0.37201	169.2	196.3	62.6	5.57	3497
3	−119.56561	−38.08399	0.59853	142.1	196.1	72.1	5.36	1519
4	−157.65877	−38.09316	0.83422	147.9	187.7	72.9	7.46	821
5	−195.74409	−38.08532	1.06207	143.0	186.3	78.7	7.10	496
6	−233.83823	−38.09414	1.29874	148.5	183.9	76.7	8.98	326
7	−271.91968	−38.08145	1.52272	140.5	179.1	83.0	8.75	227
8	−310.00802	−38.08834	1.75359	144.9	176.1	84.1	10.39	165
9	−348.09409	−38.08607	1.98219	143.4	173.8	86.2	10.31	125
10	−386.18172	−38.08763	2.21235	144.4	172.1	87.5	11.74	97
11	−424.26787	−38.08615	2.44103	143.5	169.5	88.6	11.78	77
12	−462.35529	−38.08742	2.67098	144.3	169.2	89.5	13.04	62
13	−500.44149	−38.08620	2.89971	143.5	166.2	90.5	13.19	51
14	−538.52879	−38.0873	3.12954	144.2	166.8	92.4	14.30	42
15	−576.61521	−38.08642	3.35849	143.7	163.5	92.0	14.53	36
16	−614.70241	−38.08720	3.58822	144.2	164.8	94.2	15.51	30

**Table 5:** The total energies *E_n_*, differential energies Δ*E_n_*, incremental energies Δ*E*^I^, binding energies (BE), dipole moments (*μ*), and rotational constants (RC) of C*_n_*Sb^+^ cations.

C*_n_*Sb^+^	*E_n_* (a. u.)	Δ*E_n_* (a. u.)	BE (a. u.)	Δ*E*^I^ (kcal/mol)	*μ*(Debye)	RC (MHz)
1	−43.01612		−0.23602		1.08	12013
2	−81.16886	−38.15274	0.05925	0.29527	2.68	3584
3	−119.25339	−38.08453	0.28631	0.22705	0.85	1474
4	−157.35996	−38.10657	0.53541	0.24911	2.26	835
5	−195.44758	−38.08762	0.76556	0.23014	0.52	489
6	−233.54567	−38.09809	1.00618	0.24063	1.97	331
7	−271.63483	−38.08916	1.23787	0.23169	0.43	226
8	−309.72820	−38.09337	1.47377	0.2359	1.89	167
9	−347.81792	−38.08972	1.70602	0.23225	0.48	124
10	−385.90836	−38.09044	1.93899	0.23297	0.83	98
11	−423.99872	−38.09036	2.17188	0.2329	0.65	77
12	−462.08689	−38.08817	2.40258	0.23069	1.99	62
13	−500.17791	−38.09102	2.63613	0.23355	0.87	51
14	−538.26446	−38.08655	2.86521	0.22908	2.06	43
15	−576.35619	−38.09173	3.09947	0.23426	1.11	36
16	−614.44148	−38.08529	3.32729	0.22782	2.10	31

**Table 6:** The total energies *E_n_*, differential energies Δ*E_n_*, incremental energies Δ*E*^I^, binding energies BE, dipole moments (*μ*), and rotational constants (RC) of C*_n_*Sb- anions.

C*_n_*Sb^−^	*E_n_* (a. u.)	Δ*E_n_* (a. u.)	BE (a. u.)	Δ*E*^I^ (kcal/mol)	μ (Debye)	RC (MHz)
1	−43.46493		0.21279		5.50	12153
2	−81.58147	−38.11654	0.47186	0.25908	8.89	3348
3	−119.68068	−38.09921	0.7136	0.24174	9.78	1528
4	−157.77531	−38.09463	0.95076	0.23715	13.03	800
5	−195.86996	−38.09465	1.18794	0.23718	13.52	496
6	−233.96096	−38.09100	1.42147	0.23353	16.53	321
7	−272.05265	−38.09169	1.65569	0.23422	16.93	227
8	−310.14274	−38.09009	1.88831	0.23262	19.74	163
9	−348.23223	−38.08949	2.12033	0.23202	20.16	125
10	−386.32214	−38.08991	2.35277	0.23243	22.76	96
11	−424.41010	−38.08796	2.58326	0.23049	23.26	77
12	−462.49915	−38.08905	2.81484	0.23158	25.04	62
13	−500.58704	−38.08789	3.04526	0.23042	26.30	51
14	−538.67748	−38.09044	3.27823	0.23296	28.52	42
15	−576.76336	−38.08588	3.50664	0.22841	29.33	36
16	−614.85418	−38.09082	3.73999	0.23335	31.33	30

**Table 7:** The selected structural parameters (the C-C bond adjacent to Sb and the C-Sb bond in Å), C-Sb stretching frequencies (in cm^−1^), and HOMO-LUMO gaps (in eV) for results obtained at the B3LYP/6-31G(d)/SDD and B3LYP/def2-TZVP/SDD level (denoted as normal and italic fonts, respectively) for C_4_Sb, C_5_Sb, C_6_Sb, C_10_Sb, and C_11_Sb.

	C_4_Sb	C_5_Sb	C_6_Sb	C_11_Sb	C_12_Sb
C-C	1.277 *1.267*	1.288 *1.281*	1.277 *1.267*	1.284 *1.275*	1.274 *1.263*
C-Sb	1.941 *1.941*	1.946 *1.941*	1.949 *1.949*	1.949 *1.949*	1.964 *1.966*
C-Sb Stretching frequency	378.3 *384.5*	331.3 *335.2*	291.7 *294.9*	204.8 *204.9*	190.4 *189.6*
HOMO-LUMO gap	1.27 *1.26*	1.25 *1.27*	1.17 *1.16*	1.03 *1.04*	1.01 *0.99*

**Table 8:** Valence orbital configuration of C*_n_*Sb.

*n* orbital configuration
1 (core) 1σ^2^2σ^2^1π^4^3σ^1^ 2 (core)1σ^2^2σ^2^3σ^2^4σ^2^1π^4^2π^1^ 3 (core)1σ^2^2σ^2^3σ^2^4σ^2^1π^4^5σ^2^2π^3^ 4 (core)1σ^2^2σ^2^3σ^2^4σ^2^5σ^2^1π^4^2π^4^6σ^2^3π^1^ 5 (core)1σ^2^2σ^2^3σ^2^4σ^2^5σ^2^1π^4^6σ^2^2π^4^7σ^2^3π^3^ 6 (core)1σ^2^2σ^2^3σ^2^4σ^2^5σ^2^6σ^2^1π^4^7σ^2^2π^4^3π^4^8σ^2^4π^1^ 7 (core)1σ^2^2σ^2^3σ^2^4σ^2^5σ^2^6σ^2^7σ^2^1π^4^8σ^2^2π^4^3π^4^9σ^2^4π^3^ 8 (core)1σ^2^2σ^2^3σ^2^4σ^2^5σ^2^6σ^2^7σ^2^8σ^2^1π^4^9σ^2^2π^4^3π^4^4π^4^10σ^2^5π^1^ 9 (core)1σ^2^2σ^2^3σ^2^4σ^2^5σ^2^6σ^2^7σ^2^8σ^2^9σ^2^1π^4^2π^4^10σ^2^3π^4^4π^4^11σ^2^5π^3^ 10 (core)1σ^2^2σ^2^3σ^2^4σ^2^5σ^2^6σ^2^7σ^2^8σ^2^9σ^2^10σ^2^1π^4^2π^4^11σ^2^3π^4^4π^4^5π^4^12σ^2^6π^1^ 11 (core)1σ^2^2σ^2^3σ^2^4σ^2^5σ^2^6σ^2^7σ^2^8σ^2^9σ^2^10σ^2^11σ^2^1π^4^2π^4^12σ^2^3π^4^4π^4^5π^4^13σ^2^6π^3^ 12 (core)1σ^2^2σ^2^3σ^2^4σ^2^5σ^2^6σ^2^7σ^2^8σ^2^9σ^2^10σ^2^11σ^2^12σ^2^1π^4^2π^4^13σ^2^3π^4^4π^4^5π^4^6π^4^14σ^2^7π^1^ 13 (core)1σ^2^2σ^2^3σ^2^4σ^2^5σ^2^6σ^2^7σ^2^8σ^2^9σ^2^10σ^2^11σ^2^12σ^2^13σ^2^1π^4^2π^4^14σ^2^3π^4^4π^4^5π^4^6π^4^15σ^2^7π^3^ 14 (core)1σ^2^2σ^2^3σ^2^4σ^2^5σ^2^6σ^2^7σ^2^8σ^2^9σ^2^10σ^2^11σ^2^12σ^2^13σ^2^14σ^2^1π^4^2π^4^3π^4^15σ^2^4π^4^5π^4^6π^4^7π^4^16σ^2^8π^1^ 15 (core)1σ^2^2σ^2^3σ^2^4σ^2^5σ^2^6σ^2^7σ^2^8σ^2^9σ^2^10σ^2^11σ^2^12σ^2^13σ^2^14σ^2^15σ^2^1π^4^2π^4^3π^4^16σ^2^4π^4^5π^4^6π^4^7π^4^17σ^2^8π^3^ 16 (core)1σ^2^2σ^2^3σ^2^4σ^2^5σ^2^6σ^2^7σ^2^8σ^2^9σ^2^10σ^2^11σ^2^12σ^2^13σ^2^14σ^2^15σ^2^16σ^2^1π^4^2π^4^3π^4^17σ^2^4π^4^5π^4^6π^4^7π^4^8π^4^18σ^2^9π^1^

**Table 9:** The charge population and charge difference (|ΔC|) of terminal carbon atoms which are adjacent antimony (C1) and far away from antimony (C2).

Species	C1	C2	|ΔC|	Species	C1	C2	|ΔC|
C_1_Sb	−0.33			C_9_Sb	−0.76	−0.14	0.63
C_2_Sb	−0.47	−0.05	0.42	C_10_Sb	−0.80	−0.14	0.66
C_3_Sb	−0.45	−0.14	0.31	C_11_Sb	−0.77	−0.13	0.64
C_4_Sb	−0.73	−0.22	0.51	C_12_Sb	−0.80	−0.13	0.67
C_5_Sb	−0.72	−0.19	0.53	C_13_Sb	−0.77	−0.13	0.65
C_6_Sb	−0.80	−0.18	0.62	C_14_Sb	−0.80	−0.13	0.67
C_7_Sb	−0.75	−0.15	0.60	C_15_Sb	−0.78	−0.13	0.65
C_8_Sb	−0.80	−0.15	0.65	C_16_Sb	−0.80	−0.13	0.67

## Data Availability

Not applicable.
